# High-Throughput ORB Feature Extraction on Zynq SoC for Real-Time Structure-from-Motion Pipelines

**DOI:** 10.3390/jimaging11060178

**Published:** 2025-05-28

**Authors:** Panteleimon Stamatakis, John Vourvoulakis

**Affiliations:** Department of Computer, Informatics & Telecommunications Engineering, International Hellenic University, Terma Magnisias, 62121 Serres, Greece; jvourv@ihu.gr

**Keywords:** real-time processing, structure from motion (SfM), feature extraction, ORB, FPGA, hardware acceleration, video processing, low latency, double data rate)

## Abstract

This paper presents a real-time system for feature detection and description, the first stage in a structure-from-motion (SfM) pipeline. The proposed system leverages an optimized version of the ORB algorithm (oriented FAST and rotated BRIEF) implemented on the Digilent Zybo Z7020 FPGA board equipped with the Xilinx Zynq-7000 SoC. The system accepts real-time video input (60 fps, 1920 × 1080 resolution, 24-bit color) via HDMI or a camera module. In order to support high frame rates for full-HD images, a double-data-rate pipeline scheme was adopted for Harris functions. Gray-scale video with features identified in red is exported through a separate HDMI port. Feature descriptors are calculated inside the FPGA by Zynq’s programmable logic and verified using Xilinx’s ILA IP block on a connected computer running Vivado. The implemented system achieves a latency of 192.7 microseconds, which is suitable for real-time applications. The proposed architecture is evaluated in terms of repeatability, matching retention and matching accuracy in several image transformations. It meets satisfactory accuracy and performance considering that there are slight changes between successive frames. This work paves the way for future research on the implementation of the remaining stages of a real-time SfM pipeline on the proposed hardware platform.

## 1. Introduction

In the rapidly evolving field of robotics and computer vision, the extraction and description of image features in real-time present both a significant challenge and a profound opportunity. The ability to process visual information on the fly is crucial for various applications such as autonomous navigation systems [1], object tracking [2], structure from motion [3,4], etc. This study addresses the critical need for efficient, real-time image feature extraction leveraging the capabilities of field-programmable gate arrays (FPGAs).

The key motivation behind this research is the development of a robust, high-speed solution for the initial stage of a structure-from-motion (SfM) system. SfM is a photogrammetric technique that interprets 3D structures from 2D image sequences, which can be significantly enhanced by improving the speed and efficiency of its feature extraction component. Traditional CPU-based systems often struggle to meet real-time performance requirements due to the computational complexity involved in the detection and description of features. FPGAs, with their parallel processing capabilities, offer a promising alternative by executing several operations simultaneously, drastically reducing the time for image processing tasks in various applications such as pattern recognition [5], robotics [6], cryptography [7], aerospace [8], etc.

Our approach involves the optimization and implementation of the oriented FAST and rotated BRIEF (ORB) algorithm [9] on the Digilent Zybo Z7020 FPGA board, chosen for its balanced mix of computational capability and affordability. By adapting the ORB algorithm to an FPGA-based architecture, we aim to significantly improve processing time without compromising accuracy. We hypothesize that a resource-efficient FPGA implementation of ORB can achieve real-time performance at full-HD resolution while preserving sufficient feature accuracy for reliable SfM reconstruction. This capability makes integration into larger, real-time SfM systems or similar embedded vision pipelines both practical and scalable.

The goal of this paper is not only to present a detailed account of a system’s architecture that meets real-time performance criteria while utilizing affordable hardware but also to contribute to the broader discourse on enhancing real-time computing techniques with programmable hardware. By demonstrating that high-performance and real-time results can be achieved with commercially accessible components, we hope to encourage wider adoption of FPGAs in various applications. This increased utilization could drive further innovation and accessibility, offering practical benefits in a wide range of industries.

### 1.1. FPGA vs. ASIC Rationale

While ASICs can in principle offer higher peak throughput and lower per-unit power, their non-recurring engineering costs (mask-set fabrication costs can reach the multi-thousand or even multi-million dollar range) and multi-week fabrication and shipping cycles make any design turnaround prohibitively expensive for prototyping, parameter tuning, or in-field updates. Moreover, our goal was to produce a live, tangible system rather than remain in the simulation phase—which alone cannot validate real-world timing, resource usage, or functional behavior. In contrast, FPGAs give us immediate, in-place reconfigurability and rapid design-space exploration—critical for refining high-framerate vision pipelines on emerging platforms. Reprogramming the same board allows for the on-site verification of timing, resource usage, and accuracy without waiting for a new silicon revision and enables iteration of both the algorithm and architecture on a timescale of hours rather than months.

#### Contributions

The contributions of this paper to the research community are summarized as follows.

- An optimized version of the ORB algorithm is presented, enabling real-time structure from motion for monocular vision. The system achieves 60 fps at full-HD resolution.
- A low-latency, double-data-rate (DDR) memory scheme is implemented for the Harris–Stephens corner detector, minimizing propagation delay in the data path.
- A comprehensive evaluation of the architecture is provided, including feature repeatability, matching retention, and accuracy—demonstrating the precision of the proposed ORB core.

## 2. Related Work

The realm of real-time image processing and feature extraction has been extensively explored in various domains, with a significant focus on improving computational speed and reliability for applications in robotics and autonomous navigation. The literature emphasizes the need for optimized feature extraction methods to support real-time processing in these systems, highlighting the direct impact of the work presented herein.

### 2.1. Feature Detection and Description Algorithms

Robust feature detection and description are critical for the accuracy and efficiency of image processing systems. Early work in this field was marked by the introduction of the Harris–Stephens corner detector [10], which laid the groundwork for subsequent advances in feature detection methodologies. Algorithms such as SIFT [11,12] and SURF [13] set benchmarks by providing high precision and robustness under various transformations. However, the aforementioned algorithms are computationally demanding and are not always suitable for real-time applications. They reserve a large part of the on-chip resources, leaving no adequate room for other functions. ORB (oriented FAST and rotated BRIEF) [9], used in the current work, offers a computationally efficient alternative, maintaining reasonable accuracy with significantly reduced resource utilization.

### 2.2. Structure from Motion (SFM) Pipelines

SfM pipelines are crucial for 3D reconstruction from sequential images, and their effectiveness is heavily dependent on the efficiency of the underlying feature extraction algorithms. Several open-source SfM implementations have gained prominence due to their robust performance and broad adoption. OpenMVG (open multiple-view geometry) [14], COLMAP [15,16], and OpenSfM [17], all introduced around 2016, provide comprehensive sets of SfM features, each with distinct capabilities. OpenMVG is noted for its flexibility and modularity, COLMAP offers both a command-line and a graphical interface and is recognized for its photogrammetric capabilities, and OpenSfM leverages modern web technologies for ease of use and accessibility. Subsequently released in 2018 [18], AliceVision integrates Meshroom (a graphical front end) and has been influential due to its robust algorithms and pipeline architecture. Uniquely, Meshroom supports a “live reconstruction” feature that allows for continuous updates to the SfM model as new images are captured and added to a watched folder, providing iterative previews and enabling users to make on-the-fly adjustments for improved coverage during the shoot. However, while Meshroom can process images as they are being collected, most SfM tools, including Meshroom, are not typically capable of real-time processing on the level required for instant computational results. They often still require processing, which can take a significant amount of time before obtaining final results.

### 2.3. FPGA-Based Implementations

FPGA-based systems hold significant potential for revolutionizing structure-from-motion (SfM) applications. Although full exploitation of FPGAs in SFM pipelines is still on the horizon, a variety of novel implementations have already set the stage for doing so.

Komorkiewicz et al. [19] proposed a hardware–software co-design to implement a full structure-from-motion pipeline, verifying it via ISim and MATLAB simulations but not validating it on real hardware.

Weberruss et al. [20] implemented a Harris corner-based ORB detector on a Cyclone II FPGA—discarding the FAST criterion and fully parallelizing detection with descriptor extraction taking 256 cycles; they later extended this design on an Arria V board to include a Gaussian pyramid and multiple BRIEF instances [21]. They claimed 72 FPS at 1920 × 1080 and 488 FPS at 640 × 480 but did not disclose resource usage and noted possible feature drops if an ORB module is busy at detection time.

Fang et al. [22] built a two-scale ORB hardware accelerator on an Altera Stratix V FPGA, truncating moment values to save LUTs at the expense of minor pixel location errors after rotation. They claimed 67 FPS at 640 × 480 but provided no accuracy evaluation.

De Lima et al. [23] presented an ORB descriptor computation scheme on a Xilinx Zynq XC7Z020 FPGA, using 88 foveally distributed patch pixels to build each 256-bit BRIEF descriptor. They claimed the ability to process 50 keypoints in just 6 ms.

Kalms et al. [24] implemented heterogeneous ORB detection on Zynq with four to eight pyramid levels and a FREAK descriptor, bypassing BRIEF’s intensity-centroid step and offloading orientation to LUT-based approximations. They claimed 63 FPS on Full-HD images.

Liu et al. [25] proposed a rotationally symmetric BRIEF pattern for ORB-SLAM on Zynq, forming two eight-point Gaussian-distributed sets rotated in 32 × 11.25° steps to enable descriptor rotation via bit-string shifts. They claimed an increase in framerate of up to 31× but did not assess the accuracy of this approximation.

Sun et al. [26] introduced a streaming detection stage and a superscalar-pipelined descriptor architecture using a four-bank RAM for orientation, computing first-order moments and orientation with only additions, bit shifts, and LUTs. They claimed 108 FPS on full-HD inputs with up to 8250 features, showing strong scalability.

Taranco et al. [27] designed an ORB accelerator for self-driving cars with statically scheduled rotated-BRIEF pixel-pair access optimized by a genetic algorithm to minimize bank conflicts at any rotation angle. They claimed a spread increase of 7.8× and a 1957× power reduction versus high-end CPUs.

Vemulamati and Chen [28] presented an ORB accelerator for visual SLAM on an Avnet Ultra96 ZU3EG FPGA-SoC, parallelizing image pyramid, FAST detection, Gaussian filtering, and moment computation on a per-pixel basis and computing X/Y moments recursively from incoming/outgoing columns. They claimed speed increase of 1.55× and 1.35× over desktop CPU and FPGA baselines, respectively, while preserving SLAM accuracy.

Wasala et al. [29] proposed a real-time ORB feature extraction architecture for 4K video, reading four pixels per clock from the AXI Stream interface and detecting stable features via FAST and non-maximum suppression. They claimed 60 FPS at 3840 × 2160.

Belmessaoud et al. [30] designed an ORB extraction and matching architecture performing extraction in one clock cycle, computing patch moments with adders and shift registers, approximating orientation in 2.81° steps, and bypassing coordinate rotation via multiplexers and BRAM. They claimed a throughput of 94 Mp/s (≈163 FPS) with cross-check matching.

Zhang et al. [31] developed an ORB feature-extraction accelerator using FIFO buffers to cache descriptor data without stalling pixel streaming, blocking only when the FIFO is full. They claimed a latency of 1.4 ms for 640 × 480 images with 500 keypoints.

Qi et al. [32] described a three-level parallel ORB architecture on the ZCU104 board—levels 1–2 for detection and orientation and level 3 for pyramid construction. They claimed 108 FPS, demonstrating that fine-grained task partitioning meets high-throughput targets.

Despite the advancements in hardware–software co-design systems for structure-from-motion computation, it is noteworthy that a complete structure-from-motion pipeline has yet to be implemented and rigorously tested on actual hardware. Although simulation tools provide valuable information, they cannot fully capture deployment constraints. A complete hardware-tested SfM system remains an open challenge that requires further research.

## 3. Materials and Methods

### 3.1. Methodology

In developing an efficient solution for real-time feature extraction critical to the structure-from-motion (SfM) pipeline, our approach focuses on utilizing the oriented FAST and rotated BRIEF (ORB) algorithm. ORB was selected due to its balance of computational efficiency and robust performance [33,34,35,36], essential in real-time systems where processing speed and resource constraints are critical.

#### 3.1.1. Choice of ORB

ORB integrates the FAST keypoint detector and the BRIEF descriptor, enhancing them with rotation invariance, while these features are generally beneficial, they introduce unnecessary complexity in applications like structure from motion (SfM), where the input is essentially a video stream. The efficiency of ORB is derived from the use of a centroid-based intensity measure for corner orientation and a binary descriptor. The descriptor uses pairs of pixels selected through sampling of the dataset to ensure high variance, mean values close to 0.5, and uncorrelated pairs, significantly improving the matching process.

#### 3.1.2. Modifications for SfM

Although ORB provides numerous advantages, certain aspects of the algorithm are not required for our application within an SfM pipeline. Specifically, both the scale and rotation invariance characteristics, while generally valuable, contribute additional computational overhead that is not necessary for our purposes. In the context of SfM, the relative motion between frames is more crucial than the absolute orientation or scale of individual features. Moreover, given the high frame rate of 60 frames per second used in our specific board and real-life scenario, significant rotation or scale changes are unlikely to occur within such a brief timeframe. This rapid succession of frames minimizes the possible variation in orientation and scale between consecutive images, thus reducing the need for these computational aspects of ORB. Consequently, we propose modifications to the ORB implementation that simplify both the scale space construction and the computation of orientation. We focus solely on the most relevant scales and omit rotation invariance to achieve faster processing times while maintaining adequate detection and matching accuracy essential for SfM tasks.

#### 3.1.3. Optimization for FPGA

To fully leverage the capabilities of FPGA hardware in executing the ORB algorithm, we have optimized the algorithm by restructuring its architecture for parallel processing to exploit the parallelism inherent in FPGA architectures, which markedly reduces the latency involved in feature extraction and enhances the system’s overall efficiency for real-time applications.

### 3.2. Implementation

Hardware execution is continuous, operations are concurrent and mostly synchronized by clock pulses, and immediate data access is limited in size. Thus, porting an algorithm to hardware mandates certain modifications.

**Figure 1:**
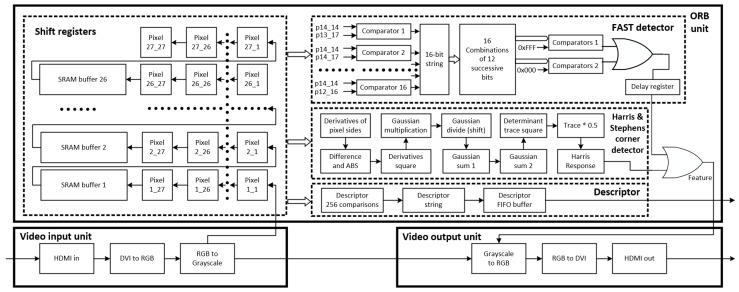
Block diagram of ORB architecture. Note: the asterisk (*) indicates multiplication.

The block diagram of the proposed architecture is depicted in Figure 1. It consists of the “Video input unit”, the “ORB unit” and the “Video output unit”. The “Video input unit” receives video from the HDMI port and converts it to grayscale. The “ORB unit” processes grayscale frames, detects features and calculates their descriptors. The “Video output unit” converts grayscale data to RGB and sends them to the HDMI output port. The video input and output units consist of more blocks than those existed in Figure 1. Blocks such as AXI4 interconnects for AXI4 master/slave interfaces, video timing controllers, DMA controllers, as well as the Zynq Processing System are also included in the architecture.

The “ORB unit” consists of a RAM-based shift registers block that holds image data of a 27 × 27 image patch around the pixel of interest. This block makes image data available concurrently to the next three blocks that are used for feature detection and description. Feature detection consists of the “FAST detector” and the “Harris & Stephens corner detector”. The outputs of both blocks are OR-ed together to produce the “Feature” indicator bit. The “Descriptor” block calculates the descriptor bit string for every pixel; however, it is outputted appropriately only when a pixel constitutes a feature.

The FAST algorithm requires information from a 7 × 7 image patch surrounding a pixel of interest, but for descriptor extraction, a 27 × 27 image patch is necessary because the farthest pixels used for the comparison are 13 positions away from the central pixel. Given that the input frames have resolution 1920 × 1080 pixels, we need 729 signals for the pixels within the window and 26 buffers of size 1894 (1920–26) in order to implement a rolling window technique.

**Figure 2:**
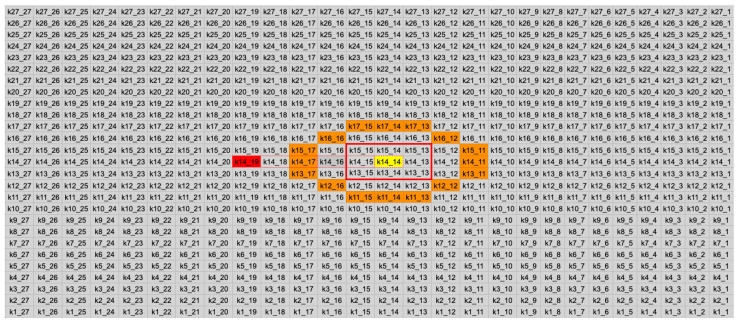
Concurrently available image patch. Yellow indicates the FAST comparison central pixel; orange highlights the Bresenham circle pixels used in the FAST comparison; the red square marks pixels used to Gaussian-weight the intensity shifts surrounding the central pixel; and solid red indicates the exported pixel.

Considering that a sliding window of 27 × 27 pixels is available at each clock cycle, as illustrated in Figure 2, the following operations are applied.

- A new pixel is inserted (k1_1).
- The FAST algorithm is applied to the central pixel of the sliding window (comparing the yellow k14_14 with the orange pixels).
- The descriptor for the central pixel (k14_14 yellow) is created.
- The pixel k14_19 (red) is checked if it constitutes a feature, and if it is, its descriptor is exported.

**Figure 3:**
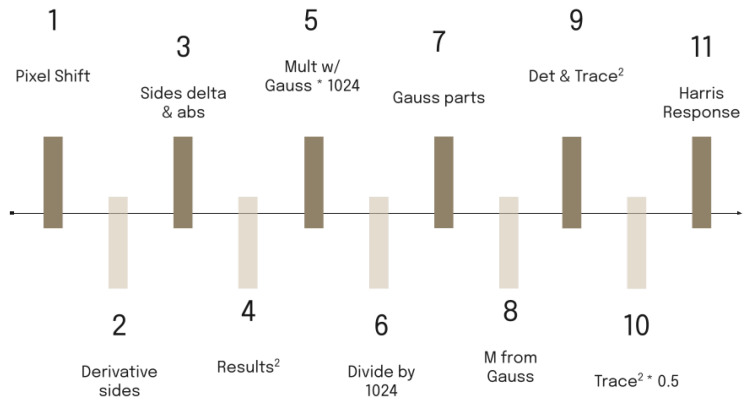
Double-data-rate clock pulse timeline. Note: the asterisk (*) indicates multiplication.

The reason for not exporting the central pixel directly after the FAST comparison and descriptor generation comes from the need to calculate its Harris response value, which finally requires five clock cycles, as depicted in Figure 3. A pure combinatorial circuit for the Harris response calculation would increase the propagation delay in the data path and therefore the maximum clock frequency would be decreased, rendering the architecture incapable of supporting 60 fps at full-HD resolution. As a workaround, pipeline stages were added and both clock edges (rising and falling) were used to launch the data for the required operations. The aforementioned DDR (double data rate) scheme reduced the required number of clock cycles to five. As a result, the pixel is outputted after five clock pulses, by which time five more pixels have entered the window, making k14_19 the central pixel.

#### 3.2.1. FAST Algorithm and Descriptor Extraction

Considering that the pixel intensities of an image patch are concurrently available, as depicted in Figure 2, the FAST algorithm and the extraction of the BRIEF descriptor occur in one clock cycle.

The FAST algorithm initially includes 16 comparisons between the central pixel and the pixels lying on the Bresenham circle. The results of the comparisons form a 16-bit string. This string is decomposed to sixteen 12-bit patterns that include all combinations of 12 successive bits. Each pattern is further compared with 0xFFF or 0x000 to identify the existence of twelve consecutive bits of one or zero, respectively. The outputs of the comparators are OR-ed together to produce the signal that indicates that the pixel satisfies the FAST criterion.

Since the BRIEF descriptor is not rotated for the current application, orientation computation and relative operations, such as moments and arctangent calculation, are not applied. Thus, 256 comparisons between pixel pairs produce the descriptor bit string without any coordinate rotation. The selected sampling geometry includes sample pairs from an isotropic Gaussian distribution from the center of the image patch as in the original paper of the BRIEF algorithm [37]. The descriptor is calculated for every pixel in the pipeline, regardless of whether it will be classified as a feature or not. The descriptor is stored in suitable FIFO buffer to be extracted a few clock cycles later as soon as the Harris criterion for the central pixel produces results.

#### 3.2.2. Harris Response Calculation

Due to complex operations, a pure combinatorial circuit can not compute the Harris response in the time between two pixels at 60 fps full HD. To resolve this, we split the Harris operations into separate functions and execute them in parallel using a pipeline.

A DDR scheme is implemented for the Harris functions, as depicted in Figure 3. Both edges of the clock are used to calculate intermediate results, reducing the number of overall cycles required. The pipeline stages for each clock edge are described as follows:

- Clock edge 1 (rising): Insertion of a new pixel in the image patch (shift the image patch by one position).
- Clock edge 2 (falling): Derivative calculation part 1. Prewitt masks are applied to each pixel within the red square (depicted in Figure 2) to calculate the partial derivatives in the x and y directions. In this step, four values are produced for each pixel. These are the sums of the pixel intensities of the column on its right and on its left as well as the sums of the pixel intensities of the rows above and below.
- Clock edge 3 (rising): Derivative calculation part 2. In this step, the difference between the sums along the x and y directions and the application of the absolute value are performed. Now, each pixel within the red square has its partial derivatives computed.
- Clock edge 4 (falling): Computation of the products $I_{x}^{2}$, $I_{y}^{2}$ and $I_{x}$·$I_{y}$, required for the calculation of the second moment matrix used in the Harris criterion, from the derivatives of the previous step. The $I_{x}^{2}$, $I_{y}^{2}$ and $I_{x}$·$I_{y}$ derivative values are calculated not only for the central pixel k14_14 but also for its neighboring pixels (within the red square). This is required when weighting the derivatives based on the distance from the center pixel by applying a Gaussian kernel.
- Clock edge 5 (rising): Multiplication of $I_{x}^{2}$, $I_{y}^{2}$ and $I_{x}$·$I_{y}$ of each pixel by the corresponding element in the Gaussian kernel. Multiplication refers to integer numbers. More details on convolution with the Gaussian kernel are provided in Section 3.2.4.
- Clock edge 6 (falling): Division of the results of the previous step by applying the appropriate number of bit shifts to the right. This step is required to produce image convolution results from a normalized Gaussian kernel.
- Clock edge 7 (rising): Gaussian sum part 1. The results of the convolution between the values of $I_{x}^{2}$, $I_{y}^{2}$ and $I_{x}$·$I_{y}$ and the Gaussian kernel for the central pixel require the addition of the nine elements of the 3 × 3 matrices obtained from the previous steps. In this step, a sum takes place for each row, producing three intermediate results.
- Clock edge 8 (falling): Gaussian sum part 2. The sum of the intermediate results of the previous step is calculated to obtain the weighted values *gk*($I_{x}^{2}$, *gk*($I_{y}^{2}$), and *gk*($I_{x}$·$I_{y}$) for the central pixel k14_14. The separation of the sum into two steps is critical since the addition of nine elements would violate the maximum propagation delay in the datapath to support 60 fps full-HD video frames.
- Clock edge 9 (rising): Calculation of the determinant and the ${\mathrm{trace}}^{2}$ of the matrix produced in the previous step.
  $$\det(M)=gk(I_{x}^{2})\cdot gk(I_{y}^{2})-gk(I_{x}\cdot I_{y})\cdot gk(I_{x}\cdot I_{y})\;{\mathrm{trace}(M)}^{2}=(gk(I_{x}^{2})+gk(I_{x}){)}^{2}$$
- Clock edge 10 (falling): Multiplication between the trace of M and 0.05 (the constant k is selected as 0.05). The operation is accomplished by approximating the 0.05 with 51/1024. First, multiplication by 51 takes place; afterwards, by shifting 10 times the product to the right, the quotient of the division is produced.
- Clock edge 11 (rising): Computation of the Harris response (R) by subtracting the result in the previous step from the determinant of M.
  $$R\;=\;\det(M)\;-\;0.05\cdot {\mathrm{trace}}^{2}$$

#### 3.2.3. Feature Point Extraction

On each rising clock edge, the Harris criterion is checked by comparing the Harris response with a threshold value for the pixel k14_19 within the 27 × 27 image patch. The threshold has been configured to 10,000 for the needs of our SfM application, which is considered quite strict. Concurrently, the same pixel is checked if it meets the FAST criteria when it was in position k14_14. If both conditions are met, the pixel is classified as a feature and the descriptor is stored. An additional 1-bit signal indicates whether the current pixel is a feature point (value 1) or not (value 0). For demonstration purposes, full-HD video at 60 fps is outputted to HDMI and is displayed on a screen. The features are depicted in red, while the rest of the pixels are shown in grayscale having the same value for each color component (e.g., an 8-bit grayscale value [11100111] is outputted as [11100111 11100111 11100111] in 24-bit color).

#### 3.2.4. Hardware Friendly Floating Point Multiplication and Gaussian Convolution

For the Gaussian filtering on the second moment matrix and for the Harris response calculation, floating point multiplications take place. Such operations are resource demanding and require longer propagation delays in the data path. In the multiplications, the multiplicand is an integer since it represents a pixel intensity and the multiplier is a floating point.

In order to implement a hardware-friendly and more efficient design, the aforementioned floating point multiplications have been replaced by operations that use only fixed-point arithmetic. That means that each multiplication treats the operands as integers. When the floating-point multiplier is known in advance, it is initially expressed in the form of a fraction. The denominator and the numerator of that fraction are multiplied by a number that gives in the denominator a product which is close to a power of two. The denominator is then approximated to the power of two. The multiplication between the multiplicand and the numerator is executed first, which is an integer multiplication. Division with the denominator of the fraction is achieved by applying the corresponding number of bit shifts to the right. This principle was applied in the last step of the Harris response calculation.

Regarding convolution between the second moment matrix and a 3 × 3 Gaussian kernel, nine floating point multiplications are required. Each element of the Gaussian kernel is a floating point number since the kernel is normalized. A power of two is selected and each element of the Gaussian kernel is multiplied by that power of two. Each product is approximated to the nearest integer. The convolution takes place with the multiplications between the corresponding elements of the second moment matrix and the integer elements of the Gaussian kernel. Each product can be accumulated, and the final sum is divided by the power of two performing right bit shifts to produce the final result. A significant detail that played a crucial role in our design was the order of accumulation and division. If the accumulation of the products between the kernel and the second moment matrix elements is applied first, and the division is applied afterwards, more accurate results are produced, but larger adders are required causing longer propagation delays in the data path. If division by a power of two precedes the accumulation, less accurate results are produced, but smaller adders are required so that it shortens the propagation delay in the data path. Since the system has a strict requirement to support full-HD image resolution at 60 fps, the second approach was chosen. Otherwise, more pipeline stages should have been added in the Harris response calculation. Moreover, the selected approach saves resources from the FPGA chip by using smaller adder circuits.

Now, let us see in detail the convolution of the partial derivatives with the Gaussian 3 × 3 kernel having a standard deviation set at 1.4, operation that is applied during pulses 6 and 7. The Gaussian kernel G is defined as follows:

$$G=\left[\begin{array}{ccc} 0.09235312 & 0.11919032 & 0.09235312\\ 0.11919032 & 0.15382623 & 0.11919032\\ 0.09235312 & 0.11919032 & 0.09235312 \end{array}\right]$$

Suppose that the multiplication between the $I_{x}^{2}$ for the k15_15 pixel and the number 0.09235312 is required. Let us assume that the square of the partial derivative is 350. The product is 350 × 0.09235312 = 32.323592. The steps applied to hardware are as follows:

1. A power of two is selected and multiplied by the element of the Gaussian kernel, herein 0.09235312. In the design, the power of two used is 1024. This means 1024×0.09235312=94.56959488.
2. The product is rounded to the nearest integer, which is 94.56959488→95.
3. The multiplication performed in hardware is between 95 and the square of the partial derivative (350). Both multiplicands are integers. The product is 350×95=33,250.
4. Finally, 10 right shifts are applied to produce the result of the initial multiplication. In this case, $1024=2^{10}$, so shift_right(33,250,10) is 32.

The power of two used to multiply the initial floating point value determines the accuracy of the operation. Higher powers of two provide better precision. However, the higher the power of two, the more resources required from the FPGA. A good compromise between accuracy and the required resources is $2^{10}=1024$, which is applied to each floating point multiplication in the proposed work.

For our Gaussian kernel, each element is replaced as follows:

$$\begin{array}{cc} \; & 0.09235312\times 1024=94.56959488\to 95\\ \; & 0.11919032\times 1024=122.05088768\to 122\\ \; & 0.15382623\times 1024=157.51805952\to 158 \end{array}$$

#### 3.2.5. Room for Improvement

Since an image patch of 27 × 27 pixels is concurrently available, it is feasible to perform the 11 aforementioned operations for the Harris corner detector before the k15_15 becomes the central pixel. The required data are contained within a 7 × 7 image subwindow which is inside the initial 27 × 27 image patch. This improvement would extract a feature six clock cycles earlier than the current implementation. It would lead to a performance gain of 74 nanoseconds or else to a speed that increases for 0.0384%. Mostly, the gain would be in the reserved resources from the FPGA. At the moment, a four-position FIFO buffer has been employed to store descriptors in each clock cycle. The improvement mentioned above is considered future work and will be addressed along with the next steps of the SfM pipeline, which are discussed in Section 5.

## 4. Results and Discussion

### 4.1. Software Validation

To evaluate the robustness of the proposed architecture, we recreated the VHDL code for the optimized ORB algorithm in Python such that the Python code had the same output as the hardware. Several transformations were applied to a set of images such as Gaussian blur, brightness, contrast, Gaussian noise, rotation, scaling, shear and translation. Seven commonly referenced images (Airplane, Baboon, Barbara, Boats, Goldhill, Lena, and Peppers) were used for testing. Each transformation was applied to the initial image (called as baseline image) 30 times, gradually increasing the effect in each iteration. In the rotation test, for example, the image was artificially rotated from 1 to 30 degrees, generating 31 images in total, including the baseline. In blur transformations, Gaussian filtering was applied using a standard deviation starting from σ = 0.25 to σ = 7.50 with step 0.25. Similar reasoning was applied to the remaining transformations.

Subsequently, three metrics were calculated for each transformed image as follows:

$$\mathrm{Feature}\;\mathrm{Repeatability}=\frac{\mathrm{original}\;\mathrm{features}\;\mathrm{repeated}}{\mathrm{original}\;\mathrm{features}}$$

$$\mathrm{Match}\;\mathrm{Retention}=\frac{\mathrm{matches}}{\mathrm{original}\;\mathrm{features}}$$

$$\mathrm{Matching}\;\mathrm{Accuracy}=\frac{\mathrm{correct}\;\mathrm{matches}}{\mathrm{matches}}$$

These tests were conducted by comparing each image with both the baseline and the previous iteration in the transformation sequence. We established the following matching criteria based on the Hamming distance:

1. A match must be at least 75% better than the second-best match (Lowe’s ratio test [12]).
2. The match must be at least 75% identical (a quality threshold that we define).

**Figure 4:**
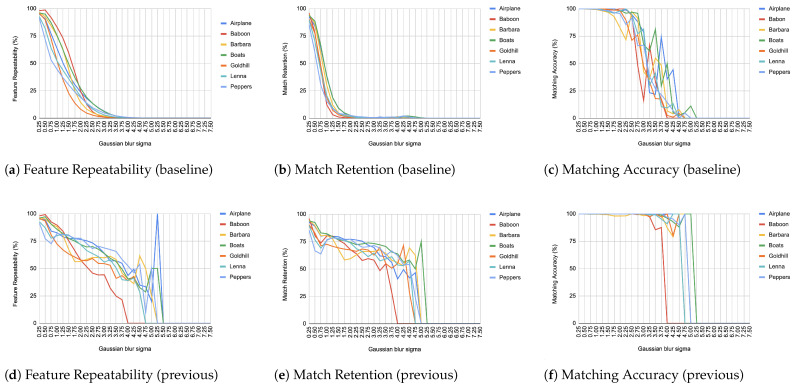
Evaluation results for Gaussian blur.

**Figure 5:**
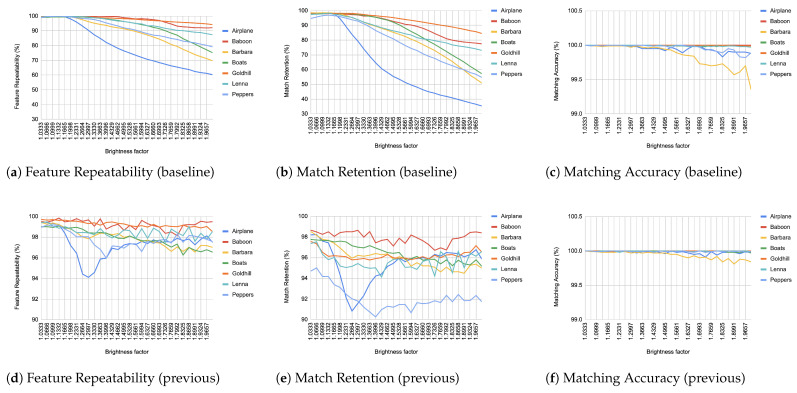
Evaluation results for brightness changes.

**Figure 6:**
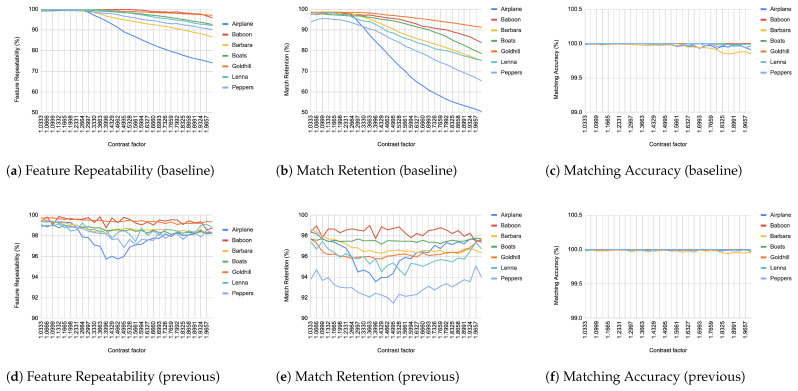
Evaluation results for contrast changes.

**Figure 7:**
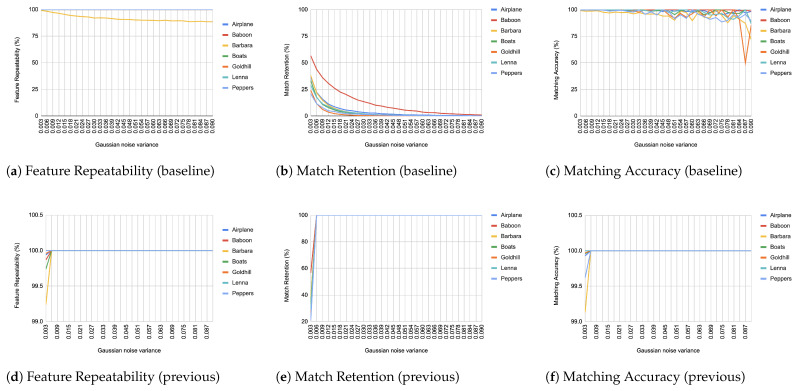
Evaluation results for Gaussian noise. (**a**) all lines but Barbara coincide at 100% and are therefore not visible (**d**–**f**) all lines coincide at 100% and are therefore not visible.

**Figure 8:**
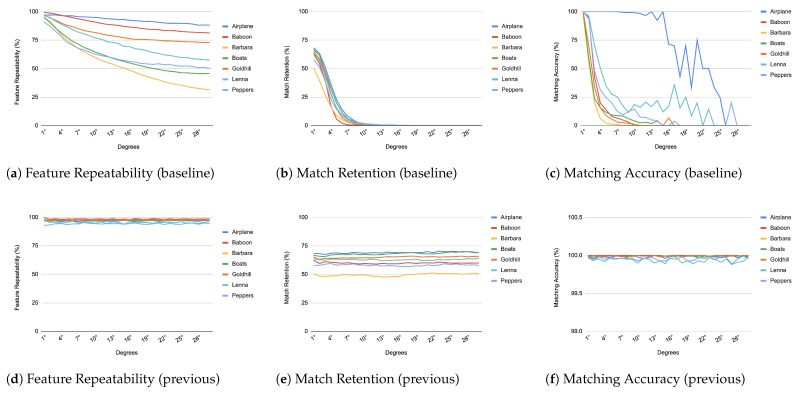
Evaluation results for rotation changes.

**Figure 9:**
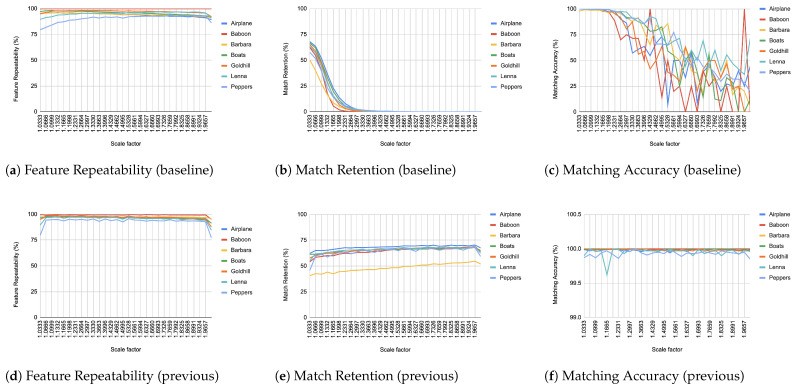
Evaluation results for scale changes.

**Figure 10:**
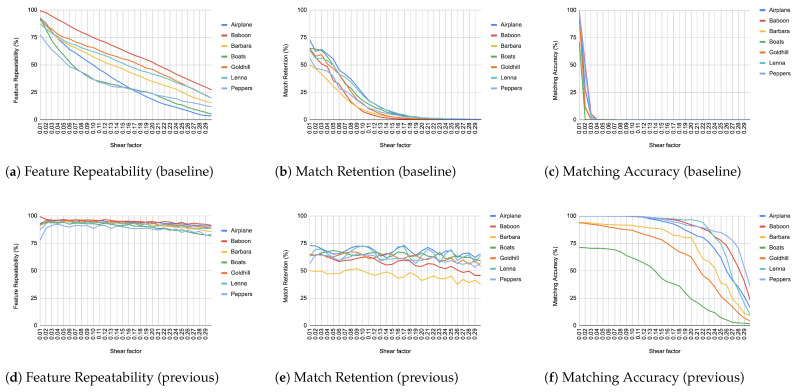
Evaluation results for shear changes.

**Figure 11:**
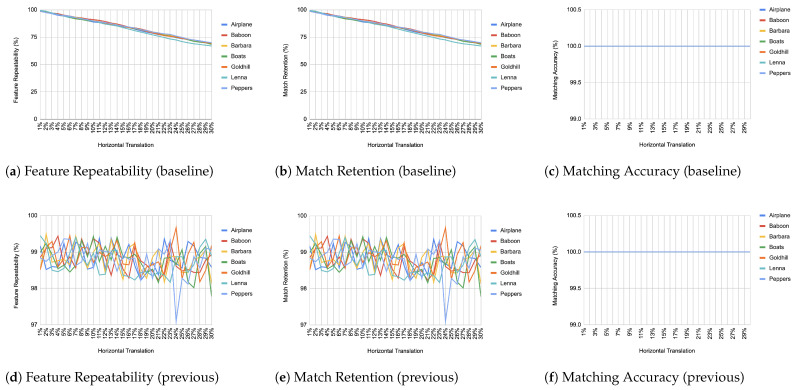
Evaluation results for translation changes. (**c**,**f**) All lines coincide at 100% and are therefore not visible.

When comparing an image from each transformation step with its immediate predecessor, we observe consistently high matching accuracy, typically in the thousands, rarely dropping below 99% in all images and transformations, as depicted from Figure 4, Figure 5, Figure 6, Figure 7, Figure 8, Figure 9, Figure 10 and Figure 11.

In contrast, when comparing transformed images to the baseline, a decrease in match retention is observed, meaning that the number of matches between features is reduced. This is especially evident in “heavy” transformations like rotation and shear. As expected, this decline occurred because we removed the intensity centroid from the calculations and the produced descriptors were not rotated to prioritize speed. The further the transformation deviated from the baseline, the more matches continued to decrease.

However, we consider these results acceptable, particularly given the “consecutive frames” nature of our input. Even in cases of rotation and shear, hundreds of feature matches persist after five to seven iterations from the baseline (equivalent to 7 degrees of rotation or a shear factor of 0.07, respectively), depending on the image. These remaining matches are sufficient for the subsequent steps in a structure-from-motion pipeline. It is worth noting that such transformations represent extreme cases. For instance, in a 60 frames-per-second setup like ours, the time between two frames is only around 16.67 milliseconds. A 7-degree rotation in such a short time frame would indicate extremely fast motion, making the primary challenge the image capture itself, rather than detection, description, or matching in the first place.

It is worth mentioning that in the blur transformation (Figure 4), for large values of the standard deviation (sigma), a decrease is observed in all metrics used in the evaluation. This is because the resulting images are excessively blurred, making object discrimination impossible. Therefore, the FAST as well as Harris criteria fail to recognize features, resulting in a corresponding reduction to repeatability, retention and accuracy. However, this constitutes an image capture issue rather than an ORB design drawback.

What was a significant inhibiting factor in the success of our implementation was the Gaussian noise transformation. The moment that noise is inserted into any kind of image, it is immediately picked up as features. The results, as depicted in Figure 7, hide pitfalls because the features that are being matched constitute just noise and do not represent reality. This means that when it comes to the reconstruction of a scene, those “fake” features will become highly problematic to deal with unless addressed beforehand. This problem resurfaced during real-world tests, as can be seen in Section 4.5.

### 4.2. Comparing Against a Widely Accepted Implementation

Feature points detected by our implementation were compared with the implementation of the ORB algorithm from the SciImage library in Python v3.12 [38]. The library’s default setting uses FAST-9, which we changed to FAST-12 to ensure stronger feature points and to match exactly the configuration of the proposed architecture. The previously mentioned set of images (Section 4.1) was used once more to allow comparison between the results of SciImage’s library and the results of our implementation.

When the FAST-12 criterion was applied, the comparison results showed that there is a match in position and number of detected features between both implementations. Slight changes to the FAST threshold and Harris configuration parameters of our implementation produce fewer or more features depending on how strict the criteria have become. When the comparison is performed using the FAST-9 configuration in the SciImage library, then the features produced in the software library are more, as expected.

### 4.3. Hardware Validation

The resulting implementation ensures real-time performance for feature extraction and descriptor computation, achieving a per-frame latency of 192.7 µs when processing a 60 FPS input stream at 1920 × 1080 resolution on the Digilent Zybo Z7020 FPGA board. The input video is streamed via the board’s HDMI input port, processed in real time, and output through the HDMI port at the same frame rate (60 FPS), demonstrating seamless throughput matching the input specifications.

**Figure 12:**
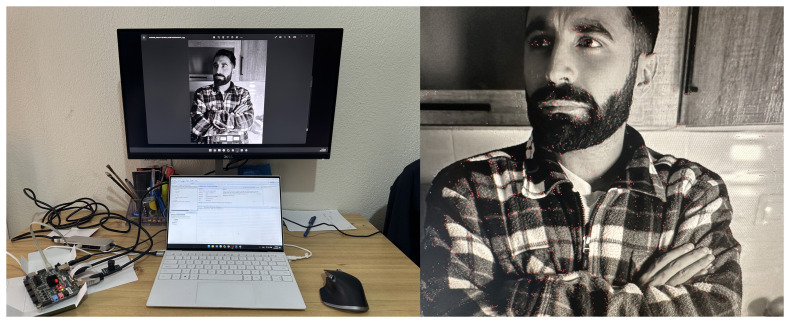
Implementation setup on the (**left**) and resulting key points (red pixels) on the (**right**).

To validate functional correctness, we designed a simplified test setup using an external monitor connected to a laptop. As illustrated in Figure 12, the FPGA board was inserted between the laptop and monitor: The laptop’s HDMI output was routed to the board’s HDMI input, while the board’s output drove the external monitor. This configuration allowed the FPGA to process the laptop’s extended desktop display in real time. Feature points detected by the algorithm were highlighted in red on the output monitor, enabling direct visual verification of detection accuracy (Figure 12).

**Figure 13:**
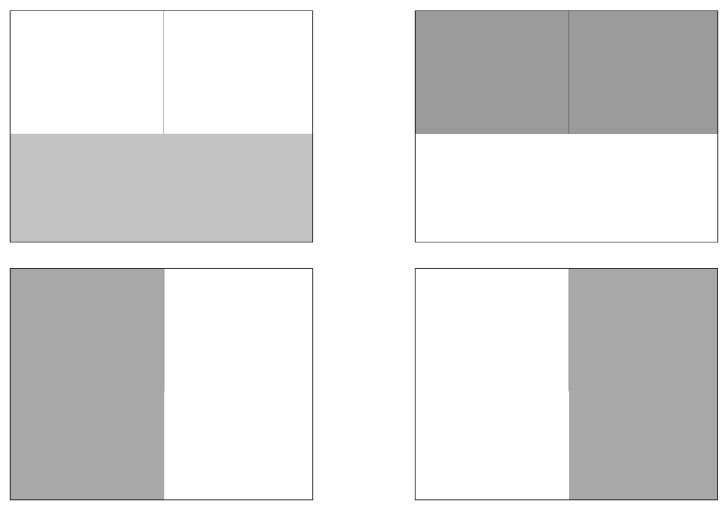
Simple examples of descriptor validation images.

**Figure 14:**
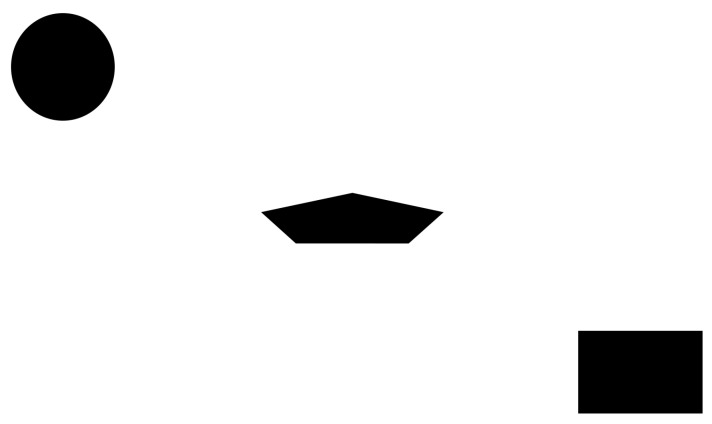
Complex descriptor validation composite image with geometric shapes.

For quantitative validation of descriptor integrity, we created five 1920 × 1080 synthetic test images (Figure 13 and Figure 14) using image editing software. For the initial four, each image contained a single vertical black line (1-pixel width) spanning from the top edge to the center. The images varied in brightness across quadrants, with the origin (0,0) defined at the image center. The fifth image contained three black-filled geometric shapes. All five images were displayed in full-screen mode on the external monitor to ensure pixel-perfect alignment between the digital image and physical display.

**Figure 15:**
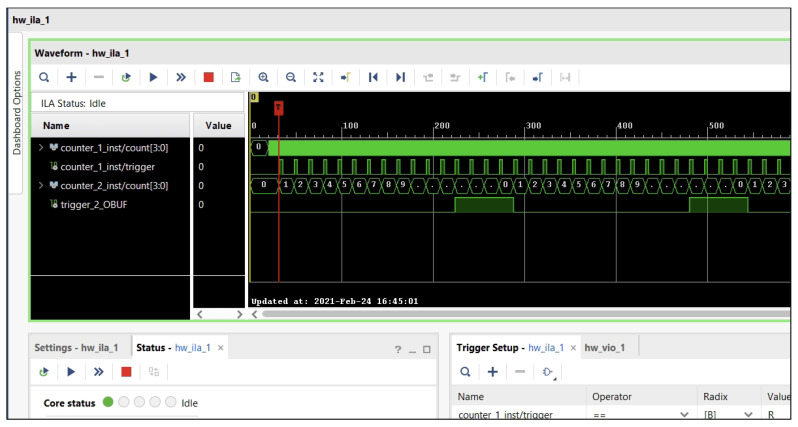
Vivado’s “Integrated Logic Analyser” (ILA) block.

Descriptor verification leveraged Xilinx Vivado’s Integrated Logic Analyzer (ILA) IP block (Figure 15). Validating the initial four synthetic test images was straightforward using the ILA’s single-trigger setup—each image contained exactly one feature point, ensuring deterministic descriptor capture via the existing ILA method. For the fifth composite test image containing multiple feature points, we implemented a per-frame feature counter trigger: the counter resets at the start of each frame and increments on every detection, firing the ILA when it matches the specified feature index. Captured descriptor values, along with the feature’s X,Y coordinates, were then compared against reference outputs from the SciImage Python library’s ORB implementation.

Experimental results confirmed the functional correctness of our FPGA implementation. Descriptor outputs from the ILA matched the SciImage reference values exactly across all test cases. This validation confirms that the feature detection and descriptor computation pipeline on the Digilent Zybo Z7020 operates as designed, with no loss of accuracy compared to the software baseline.

### 4.4. Resource Usage

**Figure 16:**
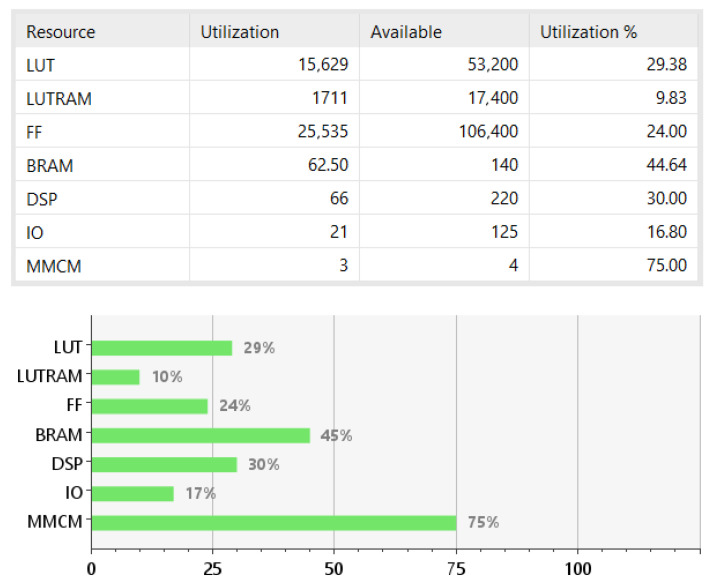
Utilization results.

In Figure 16, the resource usage of the FPGA is presented. As can be seen, less than 30% of the LUTs, FFs, and DSPs of the Zynq chip have been reserved for the proposed implementation. BRAM utilization reaches 45% since large buffers that store 26 image rows of 1920 pixels each are required. The current usage of resources let the larger part of the chip unoccupied. This is quite encouraging since there are still pending steps for the SfM process to be implemented completely.

### 4.5. Video Input from Camera and the Noise Issue

The proposed work assumes video input from the HDMI port of the board. The video was sent from the laptop. When high-quality video fed the input, the architecture performed well. However, in a real scenario, the input video usually comes from small-size camera modules attached to the system. Those cameras are prone to noise and generally give mediocre video quality.

In order to test the proposed system under more realistic conditions, the Pcam 5C camera module was employed, and the camera output was connected to the video input. The results are quite interesting, revealing some difficulties and the complexity of the overall task. In the case of video input from a camera, especially in non-optimal shooting environments, like low-light situations, we noticed in practice what was discussed during the software validation subsection. Noise was detected as features at many points in an image. Therefore, the performance of our implementation was degraded when noise was present. This can be addressed mostly in two ways, each having its advantages and disadvantages as follows.

The first way is by increasing the FAST threshold value. In our implementation, the FAST threshold has been defined to “1”, which is generally considered low but sufficient for high-quality video input. However, the appearance of noise tends to meet the criteria of both FAST and Harris Response, resulting in false feature points throughout the image. The higher we raise the FAST threshold value, the fewer false feature points we observe. However, the drawback here is that we also lose a significant number of real feature points. The higher the threshold value, the more intense the color changes around a feature point must be for it to be detected.

The second way is to remove noise using a Gaussian filter before video frames feed the core of the architecture. For evaluation purposes, we applied a 5 × 5 Gaussian kernel with σ = 1.4 to the 16 pixels of the FAST perimeter plus the central pixel used for comparison. This significantly reduced false feature points but did not eliminate them entirely. This leads to the conclusion that an adequate Gaussian filter to remove noise should be larger than 5 × 5 or have larger standard deviation or both.

The insertion of a Gaussian filter at the input of the ORB core is necessary, however, it would add more latency in the detection and description time. Moreover, it would require row buffers to parallelize its implementation and would reserve more LUTs and FFs as well as BRAM blocks. Since the FPGA resources are finite and the next steps of the SfM algorithm would also demand a significant part of the FPGA, the Gaussian filter design should be carefully tuned in order to save resources. This leads to a compromise between the efficiency of the filter and the resources used from the FPGA.

## 5. Future Work

An evaluation of the high-speed limits of the implementation will take place. We will systematically vary inter-frame rotation and scale at 60 fps to pinpoint the maximum motion our simplified, no-rotation/scale descriptor can tolerate in real time. Next, we will integrate the rBRIEF descriptor and a multi-scale pyramid—employing rotation and scale compensation—and benchmark online processing at various resolutions (e.g., 600 × 800, 800 × 1200) on the Zybo Z7020 SoC. This combined study will reveal the motion threshold and pinpoint the optimal combination of scale-pyramid levels and image resolution at which the rBRIEF descriptor can be integrated within the available FPGA, all while ensuring sufficient remaining FPGA resources for subsequent stages of SfM.

Accordingly, building on the completed feature detection and description stages, future work will focus on optimization and on the following components of the structure-from-motion (SfM) pipeline:

1. Feature matching.
2. Geometric verification.

Feature matching constitutes a computationally demanding operation. In general, each feature of the current image is compared with all feature descriptors from the previous image. A match is registered when the Hamming distance is minimized and when it is found to be less than a threshold value as well.

The special case of handling consecutive frames rather than random images significantly assists in addressing the matching problem. Since consecutive images arrive at a rate of approximately 60 frames per second, the distance between two features in successive frames that represent the same point to a scene cannot exceed a certain radius. Therefore, the search for matches can be limited to an area that does not exceed this radius. The suitable radius is under investigation and will be determined by experimentation.

An additional challenge in real-time matching arises from the variability in the total number of features per frame and how this can be managed using finite-sized hardware. One potential solution for this would be to place a grid over the image and extract only one feature point per grid cell. The extracted point will be perhaps the first one detected or the one with the strongest Harris response per grid cell. This creates a two-dimensional index that allows a very quick determination of whether the current feature was also present in the previous frame. Moreover, the number of stored features of each frame is kept low and upper bounded.

Geometric verification includes the estimation of a model that maps a sufficient number of points in common between two images. Epipolar constraints or robust estimation algorithms, such as RANSAC, are often used to reject false correspondences between images. The implementation of such algorithms requires a significant number of resources of the FPGA chip [39]. Since our intention is to maintain only a few and the strongest matches, we will investigate whether geometrical verification can be omitted, assuming that the identified matches are all true ones.

## 6. Conclusions

In this paper, an ORB feature extraction architecture optimized for structure-from-motion pipeline is presented. The architecture is fully parallelized and achieves a latency of 192.7 microseconds. The proposed system was implemented on the Zybo Z7020 board and achieved a processing rate of 60 fps in full-HD video, capable of real-time operation. In order to support such a high frame rate, the Harris corner detector was divided into 11 pipeline stages and realized using a DDR scheme. Verification of the system was successfully applied using the Vivado ILA block. Evaluation using repeatability, match retention and matching rate was carried out for the most commonly met image transformations. The proposed architecture produced satisfactory results for the target application considering that in consecutive video frames, the transformation effect has a small impact on the images.

## Data Availability

The raw data supporting the conclusions of this article will be made available by the authors on request.

## Abbreviations

The following abbreviations are used in this manuscript:

AXI4	Advanced eXtensible Interface
BRAM	Block RAM
BRIEF	Binary Robust Independent Elementary Features
rBRIEF	Rotated Binary Robust Independent Elementary Features
CPU	Central Processing Unit
DDR	Double Data Rate
DMA	Direct Memory Access
DOAJ	Directory of open access journals
FAST	Features from Accelerated Segment Test
FIFO	First-In, First-Out
FPGA	Field Programmable Gate Array
fps	frames per second
HDMI	High-Definition Multimedia Interface
ILA	Integrated Logic Analyzer
IP	Intellectual Property
LUT	Look-Up Table
MDPI	Multidisciplinary Digital Publishing Institute
ORB	Oriented FAST and rotated BRIEF
RANSAC	Random Sample Consensus
SfM	Structure from Motion
SLAM	Simultaneous Localization And Mapping
SoC	System On a Chip
UHD	Ultra-High Definition
VHDL	VHSIC Hardware Description Language
VHSIC	Very-High-Speed Integrated Circuit
